# Insecticidal and Sublethal Effects of *Artemisia scoparia* Essential Oil on *Liriomyza sativae*

**DOI:** 10.3390/insects17020170

**Published:** 2026-02-04

**Authors:** Sicheng Zuo, Rui Zhang, Bin Yan, Yuze Zhang, Zheng Duan, Jingyi Sun, Haibin Yuan, Xing Huang

**Affiliations:** College of Plant Protection, Jilin Agricultural University, Changchun 130118, China; jlauzsc1030@163.com (S.Z.); rzhang0131@163.com (R.Z.); yanbin991010@163.com (B.Y.); zhangyuze0346@163.com (Y.Z.); dz@mails.jlau.edu.cn (Z.D.); 16604447236@163.com (J.S.)

**Keywords:** *Liriomyza sativae*, *Artemisia scoparia*, chemical composition, fumigant toxicity, population parameters

## Abstract

*Liriomyza sativae* is a serious pest of horticultural and ornamental crops worldwide. The overuse of chemical insecticides has caused resistance and environmental pollution, demanding alternative control methods. Plant essential oils, with their insecticidal activity, serve as promising natural alternatives to synthetic insecticides. This study characterized the chemical composition of *Artemisia scoparia* essential oil using GC-MS and evaluated its insecticidal and growth inhibitory effects against *L. sativae*. The results showed that the major components were agropyrene (18.96%), o-cymene (12.60%), and caryophyllene oxide (11.35%). The essential oil of *A. scoparia* demonstrated significant fumigant toxicity against *L. sativae* adults, with an LC_50_ value of 0.40 µL/L air after 8 h of exposure. Sublethal concentrations (LC_10_ and LC_20_) prolonged the pre-adult developmental stages and reduced the longevity of female adults. The oviposition period and fecundity were significantly reduced compared to the control group. Additionally, reproductive parameters, including the net reproductive rate (*R*_0_), gross reproduction rate (*GRR*), intrinsic rate of increase (*r*), and finite rate of increase (*λ*), were significantly lower than the control group. The results indicate that *A. scoparia* essential oil has potent fumigant and growth-inhibitory effects on *L. sativae*, offering a promising and sustainable alternative to chemical insecticides for pest control in agriculture.

## Simple Summary

*Liriomyza sativae* Blanchard (Diptera: Agromyzidae) is a major pest affecting horticultural and ornamental crops globally. Over-reliance on chemical insecticides has led to resistance and environmental concerns, highlighting the need for alternative control methods. This study explores the insecticidal potential of *Artemisia scoparia* essential oil, known for its potent insecticidal properties. GC-MS analysis identified the primary components of the oil, including agropyrene, o-cymene, and caryophyllene oxide. The essential oil demonstrated significant fumigant toxicity against *L. sativae*, with an LC_50_ value of 0.40 µL/L air after 8 h of exposure. Additionally, sublethal concentrations prolonged the developmental stages of the pest and reduced the longevity and reproductive rates of female adults. These results indicate that *A. scoparia* essential oil effectively inhibits the growth and development of *L. sativae*, making it a promising, eco-friendly alternative to chemical insecticides. This research provides a sustainable strategy for controlling this pest, contributing to safer and more environmentally friendly pest management practices in agriculture.

## 1. Introduction

*Liriomyza sativae* Blanchard (Diptera: Agromyzidae) is a global pest of vegetables and ornamental plants, characterized by high fecundity and short generation time [1,2,3]. The females puncture the leaves to suck leaf sap and oviposit within these punctures. The hatched larvae feed on leaf tissue, forming tunnels on the leaf surface that can reduce the photosynthetic capacity of the plant, and even cause death of the whole plant in severe cases [4,5,6]. This pest is responsible for yield losses of up to 15% in melon (*Cucumis melo*) in the Rio Grande do Norte state [7]. Presently, chemical control is the primary method used to mitigate its damage both domestically and internationally [8]. Most importantly, the long-term and frequent use of certain insecticides has led to significant resistance to multiple insecticides among pests [9,10,11]. For instance, *L. sativae* has developed 34.53-fold resistance to the new insecticide chlorantraniliprole [12]. Consequently, there is an urgent need for alternative strategies that are both eco-friendly to delay the development of insecticide resistance.

Plant essential oils (EOs) represent a compelling alternative in this regard. Essential oils are secondary metabolites produced in plant metabolism, rich in monoterpenes, sesquiterpenes, and phenylpropanoids [13,14]. They possess diverse insecticidal bioactivities and are capable of disrupting critical physiological homeostasis and interfering with the metabolic pathways of arthropod pests [15,16,17,18,19,20]. *Artemisia scoparia* is an aromatic perennial herb commonly found in Asia and Europe [21]. The essential oil from *A. scoparia* exhibits fumigant, contact, and repellent actions against a range of pests. For example, it exhibits both repellent and larvicidal activity against *Aedes aegypti* females [22]. Furthermore, it exhibits toxicity towards stored-product pests, specifically *Callosobruchus maculatus*, *Sitophilus oryzae*, and *Tribolium castaneum*, with LC_50_ values of 1.46, 1.87, and 2.05 μL/L air, respectively [23].

While traditional toxicological assays prioritize acute lethal effects, these metrics frequently underestimate the broader demographic consequences of botanical insecticides. In contrast, the age-stage, two-sex life table provides a more robust framework for evaluating sublethal effects, as it integrates survival, development, and fecundity to project long-term population dynamics. For example, Braga et al. (2025) used the age-stage, two-sex life table methodology and found that treatment with *Melaleuca alternifolia* essential oil significantly reduced the intrinsic growth rate of *Tuta absoluta* populations, demonstrating its potential for population suppression [24]. Shirvani et al. (2023) employed the same methodology to demonstrate that sublethal concentrations of *Rosmarinus officinalis* essential oil significantly affected the biological and population growth parameters of *Amblyseius swirskii* [25]. Such a comprehensive evaluation is essential for determining the efficacy of *A. scoparia* essential oil as a sustainable tool within Integrated Pest Management programs (IPM).

Research regarding the insecticidal effects on the *L. sativae* is currently scarce, and studies on its sublethal effects are also rarely reported. In this context, the present study therefore aims to investigate the components of *A. scoparia* essential oil and evaluate its insecticidal activity against *L. sativae*. We will also use the age-stage, two-sex life table methodology to explore the impact of the essential oil on the growth and development of *L. sativae*. These findings lay a scientific foundation for the development of effective and eco-friendly control strategies against *L. sativae*.

## 2. Materials and Methods

### 2.1. Insect Rearing

*Liriomyza sativae* were initially collected from a greenhouse at Jilin Agricultural University in Changchun, Jilin Province, China (43.8091° N, 125.3991° E). Insect rearing methods were slightly modified from Araujo et al. [7]. The insects were kept in screen cages (50 × 50 × 50 cm) and fed with 10% honey water. Kidney bean (*Phaseolus vulgaris* L.) with cotyledonary leaves (10–14 days) that had never been exposed to insecticides were offered as oviposition substrate. Plants were then placed in an incubator (25 ± 2 °C, 40 ± 5% relative humidity, and 16:8 h light/dark photoperiod).

### 2.2. Plant Materials and Essential Oil Extraction

The aerial parts of *A. scoparia* were collected in September 2023 in Changchun, China (43.8170° N, 125.3235° E). The fresh parts were cut into pieces and then dried in the shade for 10 days. Dried plant material (1.5 kg) was soaked in distilled water for 12 h, followed by hydrodistillation for 2.5 h using a Clevenger-type apparatus (Shanghai Yuyan Machinery Equipment Co., Ltd., Shanghai, China). The obtained essential oil was stored in amber glass vials at 4 °C until further analysis.

### 2.3. GC–MS Analysis

The essential oil analysis was performed simultaneously using GC-MS systems. A gas chromatography system (Agilent 6890N, Agilent Technologies Incorporated, Santa Clara, CA, USA) was used for gas chromatography analysis with an HP-1 capillary column (30 m × 250 μm × 0.25 μm) at a maximum temperature of 260 °C. The initial temperature was initially set at 45 °C for 3 min and then increased to 80 °C at 5 °C/min. The temperature was then raised to 240 °C at 10 °C/min. The solvent added is dichloromethane, in a ratio of 20:1. The injection volume was 0.5 µL and the gas protector was loaded with helium at a flow rate of 15.0 mL/min, with a 50:1 split ratio.

The mass spectrometer (Agilent 5975N, Agilent Technologies Incorporated, Santa Clara, CA, USA) was produced using an electron ionization (EI) at 70 ev. The ion source was operated at 230 °C and scanned in the range of 20–800 *m*/*z*. The relative content of each component was determined by calculating the retention indices and searching the NIST mass spectrometry standard library using the area normalization method.

### 2.4. Toxicity Bioassay

To assess the insecticidal activity of the essential oil of *A. scoparia*, insect mortality was determined through fumigant and larvicidal bioassays.

#### 2.4.1. Fumigant Activity Bioassay

Fumigant toxicity was assessed on two-day-old adults of *L. sativae*. Essential oil from *A. scoparia* were diluted to five concentrations of 0.16, 0.33, 0.50, 0.66, and 0.83 μL/L air with acetone (Beijing Chemical Industry Group Co., Ltd., Beijing, China, 99.5% purity), and acetone treatment was used as the control. Ten µL of samples of each concentration was placed on filter paper (8 × 1.5 cm), which was placed in a 60 mL glass bottle with 20 adults, and the solvent evaporated after 15 s. The wide-mouth bottle was sealed immediately to form a sealed chamber. All experiments were performed in incubators (25 ± 2 °C, 70 ± 5% RH, 16 L:8 D) for observation at 1, 2, 4, 6, and 8 h. Each concentration treatment was replicated three times. The adults were considered dead if they were unresponsive when the bottles were shaken.

The mortality and adjusted mortality rates were calculated using the following equations:

(1) $$M=\frac{N_{1}}{N_{2}}\times 100$$

(2) $$CM=\frac{MR_{2}-MR_{1}}{1-MR_{1}}\times 100$$

where *M*: mortality rate (%)*, N*_1_: number of dead insects, *N*_2_: total number of insects in each treatment, *CM*: corrected mortality rate (%), *MR*_1_: the mortality rate of the control group (%), and *MR*_2_: the mortality rate of the treatment group (%).

#### 2.4.2. Larvicidal Activity Bioassay

The bioassays with *L. sativae* larvae were performed by a leaf-dipping method [26]. Upon reaching the second instar stage, excess larvae were removed using an insect pin under a stereomicroscope to leave exactly 10 individuals per leaf. Treatment group leaves were dipped in five concentrations (2.00, 4.00, 6.00, 8.00 and 10.00 μL/mL) of *A. scoparia* essential oil for 5 s. The leaves used as control were treated with acetone. All treated leaves were dried at room temperature and transferred to Petri dishes (90 mm diameter) containing agar. Each treatment and control group comprised 3 leaves, with three replicates per group. The whole bioassay was maintained in incubators (25 ± 2 °C, 70 ± 5% RH, 16 L:8 D). Mortality was determined after 24 and 48 h. The larva was considered dead if it did not respond to gentle prodding with an insect needle.

### 2.5. Effects of A. scoparia Essential Oil on the Growth and Development of L. sativae

#### 2.5.1. Insect Treatment

The insects were subjected to two treatments, including the fumigation of 2-day-old adults and the leaf-dipping of second-instar larvae. Detailed procedures for each method are described below.

Concentrations of the treatment group were determined based on the LC_10_ (0.05 μL/L air) and LC_20_ (0.17 μL/L air) values from the fumigant activity bioassay, while acetone was used for the control group. After 8 h of fumigation, the surviving adults were placed in insect-rearing cages with two-leaf stage *P. vulgaris* of uniform growth condition and allowed to lay eggs.

Concentrations of the treatment group were set based on the LC_10_ (0.86 μL/mL) and LC_20_ (2.33 μL/mL) of the larvicidal activity bioassay. Acetone was used as the control. The larvae were treated using the leaf-dipping method, and each concentration was set with three replicates. The surviving larvae were reared to adulthood and then moved into insect-rearing cages with two-leaf stage *P. vulgaris* for egg laying.

#### 2.5.2. Observations and Data Recording

Oviposition of the adults was observed and the number of eggs deposited on each leaf was precisely maintained at five. The location of the eggs was marked. The hatching of the eggs was observed at regular intervals twice a day (8:30 and 20:30). After the eggs hatched, two larvae were chosen from each leaf for marking and the excess larvae were discarded to observe the development of the larvae. After the larvae reached the 3rd instar, leaves were removed and placed in Petri dishes until the larvae pupated. The pupae were separately transferred into finger-shaped tubes (outer diameter: 8 mm, length: 40 mm). The sex of the adults was recorded and numbered after their pupal eclosion. One female and one male were placed in the insect cage to pair them up. One *P. vulgaris* was placed in each insect-rearing cage and supplemented with 10% honey water, and was replaced daily. The survival and egg laying of adults were recorded. Each treatment had at least 15 pairs and the process was repeated three times.

### 2.6. Statistical Analysis

All bioassay data analysis were performed using IBM SPSS Statistics software (version 23.0, Chicago, IL, USA). The differences between the mortality data were statistically compared by a one-way ANOVA analysis. Tukey’s test was used to compare significant differences between treatments. The toxicity regression equation, the sublethal concentration (LC_10_, LC_20_), and lethal concentration values (LC_50_) were calculated using log-probit analysis. The figures were plotted by using GraphPad Prism 9.5 (GraphPad Software, Boston, MA, USA).

The life table data of *L. sativae* were processed using the computer program TWOSEX-MS Chart based on the age-stage, two-sex life table theory [27]. The means and standard errors of the life table parameters were calculated according to the bootstrap method, with 100,000 resamplings [28,29]. The following life table parameters were calculated: the age-stage specific survival rate (*S_xj_*) (*x* = age, *j* = stage, same as below), the age-stage specific life expectancy (*e_xj_*), the age-specific survival rate (*l_x_*), the age-specific fecundity (*m_x_*) and the age-specific maternity (*l_x_m_x_*), the intrinsic rate of increase (*r*), the finite rate of increase (*λ)*, the net reproductive rate (*R*_0_), the mean generation time (*T*), and the gross reproduction rate (*GRR*). Lastly, the figures were created using the Origin 2019 software.

## 3. Results

### 3.1. Analysis of Chemical Composition

The GC-MS analysis of *A. scoparia* essential oil revealed the presence of 68 compounds, representing 94.95% of the total composition of the oil. The majority components were agropyrene (18.96%), o-cymene (12.60%), caryophyllene oxide (11.35%), methyl eugenol (6.94%), capillin (5.13%), and *α*-curcumene (5.04%) (Table 1).

### 3.2. Insecticidal Assays

#### 3.2.1. The Fumigant Activity of *A. scoparia* Essential Oil Against *L. sativae*

Significant differences were observed in the corrected mortality rates after 1 h (*F* = 32.591, *p <* 0.001), 2 h (*F* = 114.368, *p <* 0.001), 4 h (*F* = 270.519, *p <* 0.001), 6 h (*F* = 184.927, *p <* 0.001) and 8 h (*F* = 264.430, *p <* 0.001) of treatment with essential oils at different concentrations.

**Figure 1:**
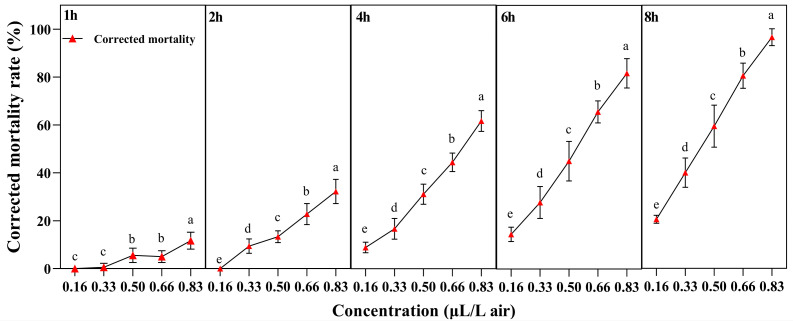
Corrected mortality of *L. sativae* adults after fumigation with *A. scoparia* essential oil. Different lowercase letters indicate significant differences (one-way ANOVA, Tukey’s Honestly Significant Difference, HSD test, *p* < 0.05) in corrected mortality among various concentrations at the same treatment time. Values labeled with the same letter are not significantly different. Data are presented as mean ± SEM (n = 9).

At the same treatment time, the corrected mortality rate of *L. sativae* had a significant increase with the concentration of *A. scoparia* essential oil. The corrected mortality rate of adults was 94.44% at the concentration of 0.83 μL/L air after 8 h treatment (Figure 1).

The lethal concentration of adults gradually decreased with longer treatment time. The LC_50_ values were 0.71 µL/L air, 0.53 µL/L air, and 0.40 µL/L air after exposure to 4 h, 6 h, and 8 h, respectively (Table 2).

#### 3.2.2. The Larvicidal Activity of *A. scoparia* Essential Oil Against *L. sativae*

Significant differences were observed in the corrected mortality rates after 12 h (*F* = 156.442, *p <* 0.001) and 24 h (*F* = 236.656, *p <* 0.001) of treatment with essential oils at different concentrations.

**Figure 2:**
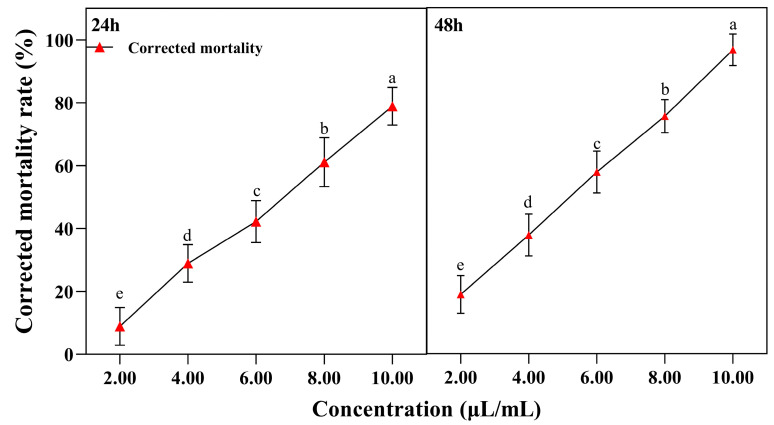
Corrected mortality of *L. sativae* larvae treated with *A. scoparia* essential oil using the leaf-dipping method. Different lowercase letters indicate significant differences (one-way ANOVA, Tukey’s Honestly Significant Difference, HSD test, *p* < 0.05) in corrected mortality among various concentrations at the same treatment time. Values labeled with the same letter are not significantly different. Data are presented as mean ± SEM (n = 9).

At the same treatment time, the corrected mortality rate of *L. sativae* significantly increased with higher concentrations of *A. scoparia* essential oil. When the treatment time was 48 h, the corrected mortality rates of larvae at 8 μL/mL and 10 μL/mL of essential oil concentrations were 75.6% and 96.7%, respectively (Figure 2).

The lethal concentration of larvae gradually decreased with the prolongation of the treatment time. The LC_50_ values of *A. scoparia* essential oil for *L. sativae* larvae were 6.76 µL/mL at 24 h and 5.14 µL/mL at 48 h of treatment (Table 3).

### 3.3. Effects of A. scoparia Essential Oil on the Development Period of L. sativae

Fumigation treatment with *A. scoparia* essential oil inhibited the growth and development of *L. sativae* offspring and reduced their adult fecundity. The larval duration, pupal duration, and total pre-oviposition period were increased under essential oil treatments compared to the control group. The oviposition days were reduced by 0.58 d and 2.12 d, respectively. Fecundity was reduced by 6.08% and 23.38%, respectively, in comparison to the control group (Table 4).

The fumigation of *A. scoparia* essential oil affected the pre-adult and adult growth and development of *L. sativae* offspring. The pre-adult stage became longer and adult longevity shorter as the concentration increased compared to the control (Table 5).

The dipping treatment with *A. scoparia* essential oil inhibited the growth and development of *L. sativae* offspring and also reduced their adult fecundity. The larval duration of offspring was extended by 0.50 days in the LC_10_ treatment and by 1.64 days in the LC_20_ treatment, as compared to the control group. The pupal duration, adult pre-oviposition period, and total pre-oviposition period were all prolonged. The adult longevity, total longevity, and oviposition days of *L. sativae* were significantly reduced. Additionally, the fecundity of the adults was significantly reduced by 27.68% and 55.04% compared with the control group, respectively (Table 6).

Dipping treatment of parental larvae significantly extended the pre-adult stage and shortened the adult longevity of female and male offspring. This impact becomes more significant as the concentration increases (Table 7).

### 3.4. Effects of A. scoparia Essential Oil on the Age-Stage Specific Survival Rate of L. sativae

The *S_xj_* were used to analyze the probability that a *L. sativae* egg born to parents treated with *A. scoparia* essential oil will survive to age *x* and develop to stage *j*. The overlapping survival rate curves of different age stages of *L. sativae* indicate that variations in developmental rates among individuals result in the coexistence of different life stages at the same time. This phenomenon leads to overlapping generations at various developmental stages.

**Figure 3:**
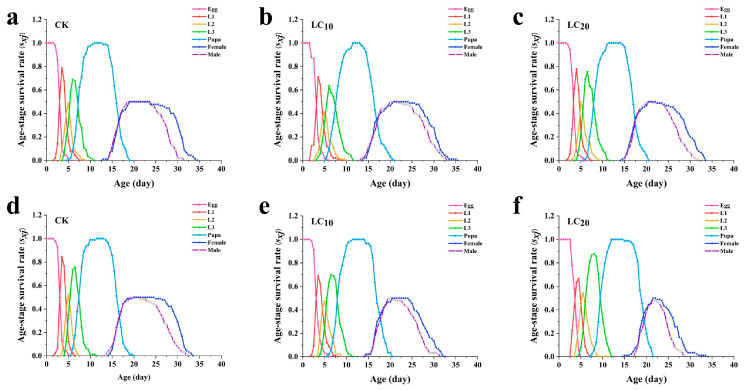
Age-specific survival rate (*S_xj_*) of *L. sativae* treated with different concentrations of *A. scoparia* essential oil. (**a**) Control group of fumigation treatment; (**b**) LC_10_ of fumigation treatment; (**c**) LC_20_ of fumigation treatment; (**d**) control group of dipping treatment; (**e**) LC_10_ of dipping treatment; (**f**) LC_20_ of dipping treatment.

Fumigation treatment with the essential oil delayed the developmental progress of the offspring compared to the control group. The survival rate of each instar generally increased first and then decreased as time increased, except for egg stage. Female adult duration was shortened when treated with LC_20_ concentration relative to the control. The highest age-stage survival rates of the 1st, 2nd, and 3rd instar larvae in the LC_10_ treated group were lower than in the control treatment (Figure 3).

Treatment of *L. sativae* using the dipping method reduced the survival rate and delayed the developmental progress of the offspring. Similar to the fumigation treatment, the survival rate of each instar of *L. sativae* offspring generally increased initially and then decreased, with the exception of eggs. Female adult duration was shortened when treated with LC_10_ concentration relative to the control group. The highest age-stage survival rates of the 1st, 2nd, and 3rd instar larvae development stages were all significantly decreased in LC_10_ treatment relative to that of the control (Figure 3).

### 3.5. Effects of A. scoparia Essential Oil on the Life Expectancy of L. sativae

**Figure 4:**
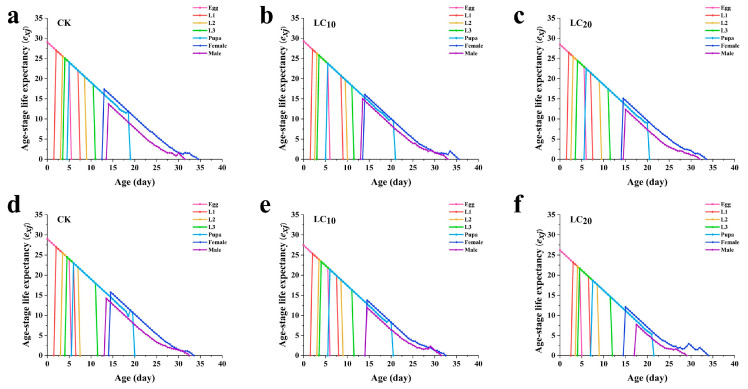
Life expectancy of each insect stage (*e_xj_*) of *L. sativae* treated with different concentrations of *A. scoparia* essential oil. (**a**) Control group of fumigation treatment; (**b**) LC_10_ of fumigation treatment; (**c**) LC_20_ of fumigation treatment; (**d**) control group of dipping treatment; (**e**) LC_10_ of dipping treatment; (**f**) LC_20_ of dipping treatment.

The life expectancy of *L. sativae* showed an overall downward trend. As the age of the *L. sativae* increased, the fumigation treatment with LC_20_ *A. scoparia* essential oil reduced the life expectancy of all stages of the offspring. The maximum life expectancy of adult males and females was significantly lower than that in the control group (Figure 4).

The life expectancy of all stages decreased significantly after treatment of *L. sativae* with LC_10_ and LC_20_ concentrations of *A. scoparia* essential oil by dipping method, which became more obvious with increasing essential oil concentration. Additionally, the maximum life expectancy of the eggs in the control group was 28.99 days, which was significantly higher than that in the treatment group (Figure 4).

### 3.6. Effects of A. scoparia Essential Oil on the Age-Specific Survival Rate and Fecundity of L. sativae

**Figure 5:**
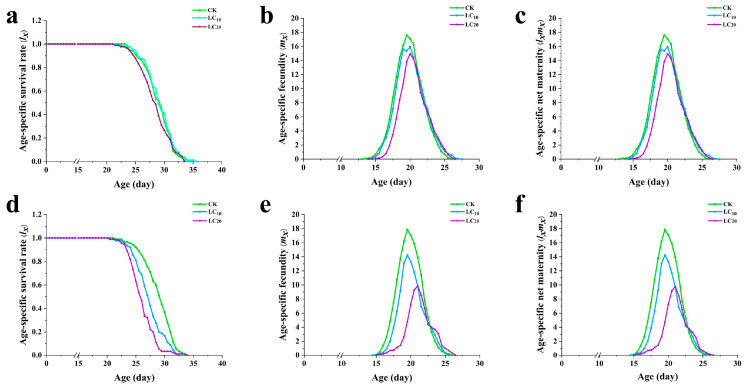
(**a**–**c**) The age-specific survival rate (*l_x_*), age-specific fecundity (*m_x_*), and age-specific maternity (*l_x_m_x_*) of *L. sativae* under fumigation treatment of *A. scoparia* essential oil; (**d**–**f**) The *l_x_*, *m_x_*, and *l_x_m_x_* of *L. sativae* under dipping treatment of *A. scoparia* essential oil.

Fumigation treatment with *A. scoparia* essential oil affected the survival and fecundity of *L. sativae*. The total life span was significantly reduced in essential oil treatments compared to the control group. Specifically, the life spans were 34.5 days for the LC_10_ treatment and 33.5 days for the LC_20_ treatment. The highest *m_x_* peaks were lower in the essential oil treatments than in the control. In addition, the maximum *l_x_m_x_* values occurred at the age 17.60, 15.96 and 14.94 days for the control, LC_10_ and LC_20_ treatments, respectively (Figure 5).

The dipping treatment with *A. scoparia* essential oil affected the survival and fecundity of *L. sativae*. The total life span of offspring was 33.50, 34.00 and 32.50 days for the control, LC_10_ and LC_20_ dipping treatments, respectively. The highest peaks for *m_x_* were lower with the LC_10_ and LC_20_ treatments than with the control. In addition, the maximum *l_x_m_x_* values occurred at the age 19.50, 19.50 and 21.00 days for the control, LC_10_ and LC_20_ treatments, respectively (Figure 5).

### 3.7. Effects of A. scoparia Essential Oil on Population Parameters of L. sativae

Fumigation treatment of *L. sativae* adults with different concentrations of *A. scoparia* essential oil influenced most population parameters of the offspring. The *λ* and *r* were significantly reduced in the LC_20_ treatment, while *T* increased. No significant difference in *R*_0_ and *GRR* were observed among the different treatments (Table 8). Different concentrations of *A. scoparia* essential oil dipping treatments resulted in decreased *r*, *λ*, *R*_0_, and *GRR*, while *T* increased (Table 9).

## 4. Discussion

The *L. sativae* is a globally significant pest of vegetables and ornamental plants, characterized by rapid reproduction and severe damage [1,2,3]. Long-term reliance on chemical control has led to the development of resistance and environmental pollution [8,9,10,11]; therefore, green pest management technologies centered on natural products such as plant essential oils have become a research focus [15,16,17,18,19,20]. In the current study, we found that *A. scoparia* essential oil exhibited potent insecticidal effects against *L. sativae*, inhibiting its growth, development, and reproduction while reducing population parameters. It is expected that the results will provide a green management strategy for *L. sativae* control.

Essential oils are composed of complex and diverse chemical components, demonstrating various biological activities against pests [30,31]. Therefore, they are not susceptible to pest resistance [32,33,34]. In this study, the chemical analysis of *A. scoparia* essential oil revealed that the main components are agropyrene (18.96%), o-cymene (12.60%), caryophyllene oxide (11.35%), and methyl eugenol (6.94%). Ickovski et al. (2020) found that agropyrene was the main compound in *A. scoparia* essential oil [35]. However, some earlier studies have indicated that the main component of *A. scoparia* oil is 1-phenyl-penta-2,4-diyne or citronellal [36,37]. Variations in the chemical constituents of essential oils could be attributed to various factors, such as climate, geography, and the conditions of cultivation, collection, and storage [38,39].

The insecticidal activity of *A. scoparia* essential oil has been demonstrated in previous studies. For instance, *A. scoparia* essential oil exhibits strong insecticidal activity against *A. aegypti* [22], *C. maculatus*, *S. oryzae* and *T. castaneum* [23]. Our results indicated that *A. scoparia* essential oil exhibited significant fumigation activity on *L. sativae* adults. Specifically, the LC_50_ value was found to be 0.40 µL/L air after 8 h exposure. Additionally, other essential oils also exhibit certain insecticidal activity against *L. sativae*. For instance, the LC_50_ values of the essential oil from *Salvia rosmarinus* were found to be 79.1 mg/L for larvae, 47.1 mg/L for adult females, and 47.8 mg/L for adult males after a 48 h treatment [40]. This study identified the main chemical components of *A. scoparia* essential oil as o-cymene, caryophyllene oxide, and methyl eugenol. Previous studies have demonstrated that these compounds exhibit significant fumigant or contact insecticidal effects against various pests. For instance, o-cymene shows good insecticidal activity against *T. castaneum* and *Liposcelis bostrychophila* [41]; caryophyllene oxide against *Dermanyssus gallinae* and *Plutella xylostella* [42,43]; and methyl eugenol against *Blattella germanica* [44]. Furthermore, although present in lower concentrations within *A. scoparia* essential oil, components such as terpinen-4-ol have been reported to exert contact toxicity against *B. germanica* and strong fumigant activity against *T. castaneum* [44,45]. In summary, the potent fumigant insecticidal efficacy demonstrated by *A. scoparia* essential oil may not be dominated by any single component, but rather results from the synergistic interaction of its multiple insecticidal chemical components. It is noteworthy that in experiments treating larvae via the dipping method, relatively high insecticidal concentrations were required. This may be attributed to the partial physical protection afforded to these leaf-boring insects by leaf tissue, limiting direct contact with the essential oil. This further indicates that the insecticidal efficacy of *A. scoparia* essential oil is closely related to its application method and the ecological habits of the target pest.

Plant essential oils not only have insecticidal activity against pests but also inhibit their growth and development [15,16,17,46,47,48]. In our study, it was observed that treatment with *A. scoparia* essential oil significantly extended the developmental period of *L. sativae* offspring. Additionally, the life span of adults was shortened. Furthermore, as the concentration of the *A. scoparia* essential oil increased, the development time of *L. sativae* larvae was extended. In addition, similar effects of plant essential oils on other insects have been reported in previous studies. For example, in treatment with LC_30_ concentration of *A. khorassanica* and *A. sieberi* essential oils, the larval developmental period of *Sitotroga cerealella* was significantly prolonged, and the adult life span of both males and females was reduced [49]. Treatment with essential oils from *A. khorassanica* and *Vitex pseudo-negundo* prolonged the larval development time of *Plodia interpunctella* while reducing its survival rate and longevity [50]. Additionally, essential oils have an impact on insect fertility. The LC_20_ concentration of *Eucalyptus camaldulensis* and *Mentha piperita* essential oils reduced the fecundity of *Trogoderma granarium* by 62.4% and 74.9%, respectively [51]. The same was also found in the treatment of *S. cerealella* with *A. khorassanica* and *A. sieberi* essential oils [49]. In this study, the longevity of female adults of the treated group was significantly lower compared to the control. Additionally, the total pre-oviposition period of females was significantly longer than that of the control group. The fumigation and dipping treatment with *A. scoparia* essential oil at LC_20_ concentration reduced the fecundity of *L. sativae* offspring by 23.36% and 55.04%, respectively. As fecundity plays an important role in population of the next generation, its reduction could suppress the population growth of the insects.

Age-stage, two-sex life table analysis is a valuable tool for understanding the population growth potential of a species in future generations. This understanding is pivotal for effectively devising Integrated Pest Management strategies [52]. In this study, *r* and *λ* of the test insects were significantly decreased when the concentration of *A. scoparia* essential oil fumigation and dipping treatments were increased. The life table parameters, especially the *r*, is the most useful parameter to evaluate the population growth potential of insect species [53]. In this study, the lower *r* value is mainly attributed to the lower survivorship, longer developmental time of immature stages, and lower fecundity of the pest. Reduction in this parameter signifies a negative impact on population growth of *L. sativae*. Similar results were reported by Borzoui et al. for *P. interpunctella* exposed to *A. khorassanica* and *V. pseudo-negundo* essential oils [50]. Sublethal concentration of *Zataria multiflora* essential oil caused a decrease in demographic parameters such as *R*_0_, *r*, and *λ* in populations of *A. swirskii* with more pronounced effects at higher concentrations [54]. Furthermore, *T* was prolonged after treatment with *A. scoparia* essential oil in this study. Similarly, treatment of *Myzus persicaev* with *Citrus limon*, *C. sinensis*, *Allium sativum*, and *Brassica nigra* essential oils resulted in a decrease in *R*_0_, *r*, and *λ*, along with a prolonged *T* [55]. The above studies have shown that essential oils have significant effect on the population parameters of pests. Although varying degrees of impact on population parameters were observed in the two treatment groups subjected to sublethal concentrations of *A. scoparia* essential oil, suggesting potential effects on F_1_ generation population growth, it is noteworthy that life table parameter analysis revealed no significant differences between the control group and the *A. scoparia* essential oil fumigation treatment group in terms of *R*_0_ and *GRR* parameters. The fumigation treatment did not significantly reduce *R*_0_ or *GRR* of offspring to the same extent as the dipping treatment. This difference may stem from differing sites of action and delivery efficiency between the two treatment modalities. Volatile compounds generated by fumigation primarily impact adult respiratory metabolism, potentially exerting limited direct effects. Conversely, the persistent systemic absorption of essential oil components through the cuticle during the larval stage is more likely to disrupt developmental programming and reproductive organ formation. This systemic stress consequently reduces the *R*_0_ and *GRR* of adults post-eclosion.

Plant essential oils hold significant potential as natural and effective alternatives to conventional pesticides. Despite their promising prospects, practical applications remain hindered by issues such as volatility and short residual efficacy. Recent research proposes encapsulating essential oils within a sodium alginate/polyethylene glycol dipropyl acrylate (SA/PEGDA) matrix [56]. This approach effectively achieves oil entrapment and controlled release, with the sustained-release period extending beyond 60 days, offering the prospect of significantly extending efficacy duration and enhancing utilization efficiency. This strategy provides a novel technical direction for formulation improvement and sustainable application of plant essential oil-based insecticides, warranting further exploration and optimization in future research.

The non-target effects of plant essential oils, particularly their impact on natural enemy insects, are crucial for assessing the safety of their application. Existing research provides reference points; for instance, *A. campestris* essential oil is considered compatible with natural enemies [57]. Experiments demonstrated that releasing parasitic wasps *Dinarmus basalis* and *Triaspis luteipes* six days after oil application still yielded parasitism rates of 13.6% and 80.3% against pests *Callosobruchus maculatus* and *Bruchus rufimanus*, respectively. Consequently, future research may further investigate the potential impacts of *A. scoparia* essential oil on non-target organisms as examined in this study.

## 5. Conclusions

In summary, the findings of this study indicated that *A. scoparia* essential oil exhibits significant fumigant activity against *L. sativae*. The results showed that the major components of *A. scoparia* essential oil were agropyrene, o-cymene, and caryophyllene oxide. Additionally, it can inhibit the growth and development of *L. sativae*; sublethal concentrations prolong the developmental stages of the pest and reduce the longevity and reproductive rates of female adults. Furthermore, key population parameters such as *r* and *λ* are also significantly reduced. From an IPM perspective, its ability to simultaneously deliver acute toxicity and impose sustained population-level suppression offers a strategic advantage. It could be developed as a component in rotation or combination with other eco-friendly tactics to manage *L. sativae* while potentially mitigating resistance development. In the future, the mechanisms of action of *A. scoparia* essential oil against *L. sativae* should be explored. Such studies will provide the necessary theoretical basis for formulating effective plant-derived insecticides based on *A. scoparia* essential oil.

## Figures and Tables

**Table 1:** GC-MS analysis of *A. scoparia* essential oil.

No	RT ^1^	Constituents	RI ^2^	Area ^3^ %
1	4.091	Cyclofenchene	1030	0.57
2	5.887	*β*-Pinene	1087	2.36
3	7.451	Myrcene	1145	0.95
4	8.254	(+)-Limonene	1176	2.11
5	8.477	1,8-Cineole	1184	1.27
6	10.299	o-cymene	1255	12.60
7	12.107	3,3,6-Trimethyl-1,5-heptadien-4-one	1332	0.14
8	13.303	(E)-myroxide	1393	0.13
9	13.468	Perillen	1401	0.09
10	15.473	Linalool	1530	0.08
11	15.940	Bornyl acetate	1563	0.13
12	16.243	Terpinen-4-ol	1585	0.11
13	16.946	(-)-Trans-pinocarveol	1639	0.14
14	17.025	(Z)-*β*-farnesene	1645	0.22
15	17.163	Selina-4(15),7(11)-diene	1656	0.22
16	17.275	Sesquisabinene A	1665	0.16
17	17.459	(-)-*α*-Terpineol	1680	0.31
18	17.729	*β*-Selinene	1701	0.24
19	17.775	*α*-Himachalene	1705	0.13
20	17.959	D(+)-carvone	1721	0.05
21	18.228	(E)-5-Isopropyl-6,7-epoxy-8-hydroxy-8-methylnon-2-one	1744	0.12
22	18.380	*α*-Curcumene	1757	5.04
23	18.465	4′-Methylacetophenone	1764	0.12
24	18.603	(-)-Myrtenol	1776	0.28
25	19.070	4(15),5,10(14)-Germacratrien-1-ol	1817	0.09
26	19.234	2-(4-Methylphenyl)propan-2-ol	1832	0.14
27	19.405	6,11-Oxido-acor-4-ene	1848	1.24
28	19.543	Neryl 2-methylbutyrate	1860	0.12
29	20.332	1,4-Dimethylazulene	1934	0.16
30	20.602	2,10-Dimethyl-9-undecenal	1960	0.18
31	20.655	*β*-(Z)-curcumen-12-ol	1965	0.22
32	20.786	Caryophyllene oxide	1978	11.35
33	20.858	2,4-Dimethyl hepta-2-dienal	1985	0.19
34	20.990	Methyl eugenol	1997	6.94
35	21.089	Benzyldiacetylene	2007	1.01
36	21.187	Nerolidol	2017	0.35
37	21.332	Humulene epoxide II	2032	1.27
38	21.983	(-)-Spathulenol	2097	0.46
39	22.101	Spathulenol	2109	3.35
40	22.220	Bisabolol oxide A	2122	0.09
41	22.515	Eugenol	2153	1.05
42	22.621	Juniper camphor	2164	0.37
43	22.765	2-Methoxy-5-prop-2-enyl-phenol	2179	0.21
44	22.864	2-Methyl-6-(p-tolyl)hept-2-en-4-ol	2190	0.57
45	23.120	*β*-Eudesmol	2217	1.20
46	23.245	Agropyrene	2231	18.96
47	23.443	Limonene glycol	2253	0.23
48	23.719	4,4-Dimethyl-tetracyclo[6.3.2.0(2,5).0(1,8)]tridecan-9-ol	2283	0.65
49	23.877	5-Hydroxy-4,4-dimethyl-1,5-diphenylpent-1-yn-3-one	2300	1.58
50	24.048	Isoaromadendrene epoxide	2320	0.29
51	24.238	*α*-Cyperone	2341	0.65
52	24.396	Alloaromadendrene oxide-(1)	2359	0.74
53	24.481	(+)-*α*-Nuciferol	2369	0.85
54	24.547	5,6,6-Trimethyl-5-(3-oxobut-1-enyl)-1-oxaspiro[2.5]octan-4-one	2376	0.19
55	24.646	Isospathulenol	2388	0.16
56	24.731	Cis-Z-*α*-bisabolene epoxide	2397	1.01
57	24.804	4-Allyl-2-methoxyphenyl 2-methylbutyrate	2406	0.81
58	25.126	3-Nitro-1-phenylheptan-1-ol	2444	0.14
59	25.211	(+)-MBF-OH dimer	2454	0.10
60	26.027	Capillin	2549	5.13
61	26.783	2-Phenylphenol	2636	0.39
62	27.493	2-Methyl-6-(4-methylphenyl)hept-2-en-1-ol	2712	0.20
63	28.085	Pentadecanoic acid	2766	0.08
64	28.223	Benzenebutanoic acid	2779	0.09
65	28.374	Isocalamendiol	2793	0.11
66	29.413	Palmitic acid	2872	0.52
67	29.617	3,4-Dimethoxybenzenepropenal	2888	0.08
68	30.301	2-Phenyl-bicyclo[2.2.1]hept-2-ene	2932	3.85

^1^ RT, retention time. ^2^ RI, retention index. ^3^ Area, peak area/total peak area.

**Table 2:** Fumigation Toxicity of *A. scoparia* essential oil against *L. sativae* adults.

Time (h)	LC_50_ (µL/L Air)	95% Confidence Interval	Regression Equation	*R* ^2^	*x* ^2^	*df*
4	0.71	0.67–0.76	*y* = 2.467*x* − 1.754	0.98	6.82	43
6	0.53	0.50–0.56	*y* = 2.949*x* − 1.560	0.97	14.91	43
8	0.40	0.37–0.43	*y* = 3.647*x* − 1.475	0.98	19.38	43

**Table 3:** Dipping Toxicity of *A. scoparia* essential oil against *L. sativae* larvae.

Time (h)	LC_50_ (µL/mL)	95% Confidence Interval	Regression Equation	*R* ^2^	*x* ^2^	*df*
24	6.76	6.26–7.31	*y* = 0.251*x* − 1.697	0.97	11.35	43
48	5.14	4.67–5.58	*y* = 0.300*x* − 1.541	0.98	13.28	43

**Table 4:** Developmental duration and fecundity of *L. sativae* treated with *A. scoparia* essential oil using fumigation method.

Stage	Control	LC_10_	LC_20_
Egg duration (days)	3.26 ± 0.06 b	3.33 ± 0.08 a	3.59 ± 0.07 a
1st-instar duration (days)	1.45 ± 0.06 a	1.50 ± 0.07 a	1.41 ± 0.06 a
2nd-instar duration (days)	0.94 ± 0.04 a	0.99 ± 0.05 a	1.02 ± 0.05 a
3rd-instar duration (days)	1.98 ± 0.07 b	2.12 ± 0.07 a	2.33 ± 0.06 a
Larval duration (days)	4.37 ± 0.10 c	4.62 ± 0.11 b	4.77 ± 0.11 a
Pupa duration (days)	8.66 ± 0.05 b	8.76 ± 0.07 ab	8.93 ± 0.06 a
Adult pre-oviposition period (days)	0.59 ± 0.05 b	0.52 ± 0.03 b	0.76 ± 0.05 a
Total pre-oviposition period (days)	16.93 ± 0.20 b	17.29 ± 0.25 a	18.00 ± 0.23 a
Adult longevity (days)	12.77 ± 0.22 a	12.54 ± 0.20 a	11.19 ± 0.24 b
Total longevity (days)	29.05 ± 0.26 ab	29.26 ± 0.26 a	28.48 ± 0.28 b
Oviposition period (days)	11.76 ± 0.13 a	11.18 ± 0.13 a	9.64 ± 0.15 b
Fecundity (eggs/female adult)	332.4 ± 9.23 a	312.16 ± 9.62 a	254.76 ± 9.05 b

Values are shown as means ± SE. Different lowercase letters in the same row indicate significant differences between treatments (*p* < 0.05).

**Table 5:** Pre-adult stage and adult longevity of *L. sativae* treated with *A. scoparia* essential oil using fumigation method.

Treatment	Pre-Adult Stage (Days)		Adult Longevity (Days)	
	Male	Female	Male	Female
Control	16.22 ± 0.18 b	16.34 ± 0.20 b	11.49 ± 0.26 a	14.04 ± 0.25 a
LC_10_	16.66 ± 0.24 a	16.77 ± 0.25 ab	11.82 ± 0.28 a	13.27 ± 0.25 b
LC_20_	17.33 ± 0.19 a	17.24 ± 0.22 a	10.02 ± 0.30 b	12.36 ± 0.28 c

Values are shown as means ± SE. Different lowercase letters in a column indicate significant differences between treatments (*p* < 0.05).

**Table 6:** Developmental duration and fecundity of *L. sativae* treated with *A. scoparia* essential oil using dipping method.

Stage	Control	LC_10_	LC_20_
Egg duration (days)	3.32 ± 0.06 c	3.48 ± 0.08 b	3.84 ± 0.06 a
1st-instar duration (days)	1.41 ± 0.04 a	1.40 ± 0.05 a	1.38 ± 0.05 b
2nd-instar duration (days)	0.89 ± 0.05 c	1.08 ± 0.06 b	1.26 ± 0.05 a
3rd-instar duration (days)	2.04 ± 0.07 c	2.37 ± 0.08 b	3.35 ± 0.09 a
Larval duration (days)	4.35 ± 0.10 c	4.85 ± 0.10 b	5.99 ± 0.10 a
Pupa duration (days)	8.74 ± 0.07 c	9.07 ± 0.09 a	9.33 ± 0.05 a
Adult pre-oviposition period (days)	0.61 ± 0.04 a	0.68 ± 0.05 a	0.71 ± 0.05 a
Total pre-oviposition period (days)	17.07 ± 0.18 b	18.02 ± 0.23 a	19.70 ± 0.22 a
Adult longevity (days)	12.58 ± 0.21 a	9.97 ± 0.22 b	7.02 ± 0.21 c
Total longevity (days)	28.99 ± 0.26 a	27.37 ± 0.26 b	26.18 ± 0.24 c
Oviposition period (days)	11.42 ± 0.11 a	8.53 ± 0.15 b	5.62 ± 0.16 c
Fecundity (eggs/female adult)	307.56 ± 10.58 a	222.42 ± 10.95 b	138.27 ± 12.99 c

Values are shown as means ± SE. Different lowercase letters in the same row indicate significant differences between treatments (*p* < 0.05).

**Table 7:** Pre-adult stage and adult longevity of *L. sativae* treated with *A. scoparia* essential oil using dipping method.

Treatment	Pre-Adult Stage (Days)		Adult Longevity (Days)	
	Male	Female	Male	Female
Control	16.38 ± 0.19 c	16.46 ± 0.17 c	11.33 ± 0.25 a	13.82 ± 0.20 a
LC_10_	17.47 ± 0.19 b	17.34 ± 0.21 b	8.98 ± 0.27 b	10.96 ± 0.29 b
LC_20_	19.33 ± 0.17 a	18.99 ± 0.20 a	5.93 ± 0.19 c	8.11 ± 0.31 c

Values are shown as means ± SE. Different lowercase letters in a column indicate significant differences between treatments (*p* < 0.05).

**Table 8:** Population parameters of *L. sativae* treated with *A. scoparia* essential oil under fumigation treatment.

Treatments	*r*	*λ*	*R*_0_	*T*	*GRR*
Control	0.26 ± 0.01 a	1.29 ± 0.01 a	166.26 ± 18.12 a	19.86 ± 0.22 b	166.55 ± 18.14 a
LC_10_	0.25 ± 0.01 ab	1.29 ± 0.01 a	156.08 ± 17.11 a	20.08 ± 0.22 ab	156.84 ± 17.13 a
LC_20_	0.23 ± 0.01 b	1.26 ± 0.01 b	127.32 ± 14.15 a	20.64 ± 0.21 a	128.61 ± 14.24 a

*r*: intrinsic rate of increase, *λ*: finite rate of increase, *R*_0_: net reproductive rate, *T*: mean generation time, *GRR*: gross reproduction rate. Different lowercase letters in a column indicate significant differences between treatments (*p* < 0.05).

**Table 9:** Population parameters of *L. sativae* treated with *A. scoparia* essential oil under dipping treatment.

Treatments	*r*	*λ*	*R*_0_	*T*	*GRR*
Control	0.25 ± 0.01 a	1.29 ± 0.01 a	153.75 ± 17.03 a	20.76 ± 0.17 b	154.33 ± 17.03 a
LC_10_	0.23 ± 0.01 b	1.26 ± 0.01 b	111.16 ± 12.91 b	21.29 ± 0.21 a	112.49 ± 12.95 a
LC_20_	0.20 ± 0.01 c	1.22 ± 0.01 c	69.13 ± 9.70 c	21.36 ± 0.28 a	72.13 ± 10.18 b

*r*: intrinsic rate of increase, *λ*: finite rate of increase, *R*_0_: net reproductive rate, *T*: mean generation time, *GRR*: gross reproduction rate. Different lowercase letters in a column indicate significant differences between treatments (*p* < 0.05).

## Data Availability

Data are contained within the article.
